# Prognostic accuracy of biomarkers of immune and endothelial activation in Mozambican children hospitalized with pneumonia

**DOI:** 10.1371/journal.pgph.0001553

**Published:** 2023-02-23

**Authors:** Núria Balanza, Clara Erice, Michelle Ngai, Chloe R. McDonald, Andrea M. Weckman, Julie Wright, Melissa Richard-Greenblatt, Rosauro Varo, Elisa López-Varela, Antonio Sitoe, Pio Vitorino, Justina Bramugy, Miguel Lanaspa, Sozinho Acácio, Lola Madrid, Bàrbara Baro, Kevin C. Kain, Quique Bassat

**Affiliations:** 1 ISGlobal, Hospital Clínic, Universitat de Barcelona, Barcelona, Spain; 2 Sandra-Rotman Centre for Global Health, Toronto General Research Institute, University Health Network-Toronto General Hospital, Toronto, Ontario, Canada; 3 Tropical Disease Unit, Division of Infectious Diseases, Department of Medicine, University of Toronto, Toronto, Ontario, Canada; 4 Department of Pathology and Laboratory Medicine, University of Pennsylvania, Philadelphia, Pennsylvania, United States of America; 5 Centro de Investigação em Saúde de Manhiça, Maputo, Mozambique; 6 Desmond Tutu TB Centre, Department of Pediatrics and Child Health, Faculty of Medicine and Health Sciences, Stellenbosch University, Cape Town, South Africa; 7 Department of Infectious Disease Epidemiology, London School of Hygiene & Tropical Medicine, London, United Kingdom; 8 ICREA, Barcelona, Spain; 9 Pediatrics Department, Hospital Sant Joan de Déu (University of Barcelona), Barcelona, Spain; 10 Consorcio de Investigación Biomédica en Red de Epidemiología y Salud Pública (CIBERESP), Madrid, Spain; Aga Khan University, PAKISTAN

## Abstract

Pneumonia is a leading cause of child mortality. However, currently we lack simple, objective, and accurate risk-stratification tools for pediatric pneumonia. Here we test the hypothesis that measuring biomarkers of immune and endothelial activation in children with pneumonia may facilitate the identification of those at risk of death. We recruited children <10 years old fulfilling WHO criteria for pneumonia and admitted to the Manhiça District Hospital (Mozambique) from 2010 to 2014. We measured plasma levels of IL-6, IL-8, Angpt-2, sTREM-1, sFlt-1, sTNFR1, PCT, and CRP at admission, and assessed their prognostic accuracy for in-hospital, 28-day, and 90-day mortality. Healthy community controls, within same age strata and location, were also assessed. All biomarkers were significantly elevated in 472 pneumonia cases versus 80 controls (p<0.001). IL-8, sFlt-1, and sTREM-1 were associated with in-hospital mortality (p<0.001) and showed the best discrimination with AUROCs of 0.877 (95% CI: 0.782 to 0.972), 0.832 (95% CI: 0.729 to 0.935) and 0.822 (95% CI: 0.735 to 0.908), respectively. Their performance was superior to CRP, PCT, oxygen saturation, and clinical severity scores. IL-8, sFlt-1, and sTREM-1 remained good predictors of 28-day and 90-day mortality. These findings suggest that measuring IL-8, sFlt-1, or sTREM-1 at hospital presentation can guide risk-stratification of children with pneumonia, which could enable prioritized care to improve survival and resource allocation.

## 1. Introduction

Pneumonia is a leading cause of illness and death in children. In 2019, pneumonia resulted in an estimated 740,000 deaths in children under the age of five, accounting for nearly 14% of all deaths in this age group [1]. Although childhood pneumonia is a global problem, it disproportionately affects low and middle-income countries.

Identifying children with pneumonia who are at high risk of severe or fatal disease is essential to reduce mortality, as such cases need early and adequate clinical management. Nevertheless, this is challenging in settings without radiographic imaging, basic or inexistent laboratory facilities, and limited qualified medical personnel. The World Health Organization (WHO) has set clinical algorithms for resource-constrained settings to diagnose pneumonia as a clinical syndrome and identify severe cases to guide patient case management [2–4]. Yet, these guidelines have limitations including subjective signs recognition. Clinical signs and laboratory measures have also been combined to generate prognostic scores for children with pneumonia or, more broadly, febrile illness [5–8]. However, these scores have not been well validated nor implemented for childhood pneumonia. New simple, accurate, objective, and inexpensive triage and risk-stratification tools are needed.

Immune and endothelial activation are implicated in the pathogenesis of severe and fatal infections. Previous studies have shown that circulating analytes of these pathways, measured at clinical presentation, have good prognostic accuracy in a range of infections, including pneumonia. They have been associated with mortality and other adverse outcomes, and they may enable improved childhood pneumonia risk-stratification [9–13]. However, only a few studies have assessed some of these biomarkers in children with pneumonia in sub-Saharan Africa [14–21].

Here we evaluated the prognostic accuracy of eight biomarkers for in-hospital and longer-term (28-day and 90-day) mortality in Mozambican children hospitalized with WHO-defined clinical pneumonia versus clinical signs and severity scores. Their biomarker levels were also compared to those in healthy community controls.

## 2. Methods

### 2.1 Study site

This study was conducted in Manhiça district, a rural area located in Maputo province, southern Mozambique. Studies conducted in the district of Manhiça have consistently described significant percentages of child hospital admissions and mortality due to pneumonia [22–24], which are exacerbated by suboptimal pneumonia management and referral practices [25]. A full description of the Manhiça population and study area can be found in previous publications [26, 27].

### 2.2 Study design

This analysis is part of a larger study designed to identify etiological biomarkers in children with respiratory symptoms [28–30]. Between 2010 and 2014, children presenting to the Manhiça District Hospital with symptoms of clinical pneumonia and fulfilling criteria for hospital admission were assessed for recruitment. Children were invited to participate in the study if they were <10 years old, had fever (≥37.5°C axillary temperature) or reported a history of fever in the preceding 24 hours, and met the 2005 WHO case definition for clinical pneumonia (cough or/and difficulty breathing plus increased respiratory rate for age) [31]. Exclusion criteria included use of antimalarials in the preceding two weeks, established or suspected tuberculosis (based on history of cough of >2 weeks or close contact with a documented case), marked hypoxemia (SpO_2_ ≤85%, as a proxy of *Pneumocystis jirovecii* infection), and participation in conflicting studies. Patients were managed in accordance with Mozambique’s national standard of care [22].

Community controls <10 years old and living in the district of Manhiça were recruited from 2010 to 2012. The Health and Demographic Surveillance System (HDSS) running in the district allowed the selection of a random sample of children within the same age group and geographic area than study patients. Controls were not considered healthy if they presented with fever, had taken any medication during the preceding 30 days, had a positive malaria microscopy exam, or showed any other important sign of disease. Human immunodeficiency virus (HIV) testing was not performed in this group.

### 2.3 Clinical, laboratory, and radiological data

Standard clinical questionnaires were completed for all subjects. Upon recruitment and prior to initiating treatment, venous blood and a nasopharyngeal aspirate were collected from pneumonia patients. Briefly, blood was used on-site for hematocrit, full blood count, blood smears for malaria diagnosis, blood culture, and HIV-1 testing. Dried blood spots were collected and used for molecular screening for *Streptococcus pneumoniae* and *Haemophilus influenzae* type B. The nasopharyngeal aspirate was used for PCR-based determination of influenza virus (A, B and C), respiratory syncytial virus (A and B), adenovirus, parainfluenza (1, 2, 3 and 4), coronavirus (229E and OC43), rhinovirus, enterovirus, human metapneumovirus, and bocavirus. Cerebrospinal fluid and pleural effusion were collected and cultured if clinically indicated. The detection of a non-contaminating bacterial organism in cerebrospinal fluid, pleural effusion, and/or blood was defined as an invasive bacterial disease. Pneumonia patients underwent anteroposterior chest radiography and X-rays were interpreted by two experienced clinicians following a WHO-designed protocol [32]. From community controls, venous blood was collected and used for blood smears for malaria diagnosis. Further details on all procedures can be found elsewhere [30].

### 2.4 Biomarker quantification

A volume of ~2 mL of the venous blood was collected in EDTA coated tubes, centrifugated, and the derived plasma was isolated and stored at -80°C until shipment to University Health Network, Canada. Plasma concentrations of angiopoietin-2 (Angpt-2), interleukin-6 (IL-6), interleukin-8 (IL-8), procalcitonin (PCT), soluble fms-like tyrosine kinase-1 (sFlt-1, also known as soluble vascular endothelial growth factor receptor*-*1 or sVEGFR-1), soluble tumor necrosis factor receptor 1 (sTNFR1), and soluble triggering receptor expressed on myeloid cells 1 (sTREM-1) were quantified using the multiplex Luminex platform with custom-developed reagents from R&D Systems [33]. Individual Luminex plates included 10% duplicates. C-reactive protein (CRP) was quantified by enzyme-linked immunosorbent assay (ELISA) (R&D DuoSet) and all assays were run in duplicate. Procedures were performed according to manufacturers’ instructions and blinded to clinical data. Biomarker concentrations outside the dynamic range were assigned a value of one-third of the lowest limit or the highest limit of the standard curve (S1 Table). If volume was insufficient for both assays, Luminex was prioritized over ELISA.

### 2.5 Post-discharge mortality data

Post-discharge mortality data were retrieved from the Manhiça HDSS. Each person living in the surveillance area is issued a unique permanent identification number, allowing HDSS data to be linked to the study database. HDSS mortality data were obtained through semestral household visits, weekly updates from key community informants, and daily hospital visits [26, 27].

### 2.6 Data analysis

All statistical analyses and graphs were performed using Stata 16.0 (Stata Corp, College Station, TX, USA). Our primary outcome was in-hospital mortality during initial admission. Secondary outcomes were 28-day and 90-day mortality. Children who were transferred to a higher-level hospital or absconded (i.e., left against medical advice) were excluded when evaluating the primary outcome, and children without information on vital status at 28 or 90 days were excluded when assessing secondary outcomes. A sensitivity analysis was conducted to evaluate 28-day and 90-day mortality excluding children who died during initial hospital admission. Biomarker concentrations in two groups were compared using Mann-Whitney U tests. Areas under the receiver operating characteristic curve (AUROCs) for one parameter were analyzed nonparametrically. Logistic models were used to estimate odds ratios (ORs) and AUROCs for multivariable models. Biomarker data were log-transformed for inclusion in logistic regression models. Models and variables were compared in terms of discrimination, by comparing AUROCs using the algorithm suggested by DeLong *et al*. [34]. Best two-biomarker combination was determined based on highest AUROC, excluding CRP from this analysis. Biomarker correlations were explored using Spearman’s rank correlation coefficients. Two-sided p-values <0.05 were considered statistically significant.

Severe pneumonia was defined, according to the WHO 2013 pocket book, as the presence of at least one of the following: oxygen saturation <90% or central cyanosis, severe respiratory distress (i.e., grunting, very severe chest indrawing), or any danger sign (i.e., inability to breastfeed or drink, lethargy or reduced level of consciousness, convulsions) [3]. This WHO severity definition, SpO_2_ [35], the Respiratory Index of Severity in Children-Malawi (RISC-Malawi) score [7, 36], and the Lambaréné Organ Dysfunction Score (LODS) [37–39] were pre-specified as important mortality prognostic factors. The RISC-Malawi score takes into account degree of hypoxemia, severity of malnutrition (here using mid-upper arm circumference [MUAC]), sex, presence of wheeze, and level of consciousness; while the LODS considers deep breathing, prostration, and coma. Biomarker cut-offs were derived for in-hospital mortality using the Youden’s index method (J = max[sensitivity+specificity-1]) and pre-specified likelihood ratios. A negative likelihood ratio (LR-) of 0.1 and a positive likelihood ratio (LR+) of 10 were used as they indicate large changes from pre-test to post-test probabilities. Lastly, we applied sTREM-1 cut-offs derived using same likelihood ratios for 7-day in-hospital mortality by Leligdowicz *et al*. [13] in a cohort of Ugandan febrile children. Corresponding sensitivity, specificity, LR-, LR+, positive predictive value (PPV), and negative predictive value (NPV) were calculated for each cut-off.

### 2.7 Ethical approval

This study was approved by the Mozambique National Bioethics Committee (reference No. CNBS-IRB00002657) and the Ethics Committee at the Hospital Clinic in Barcelona, Spain (reference No. 2010/5590), and performed in accordance with the principles of the Declaration of Helsinki. Parents or guardians provided written informed consent after a detailed explanation of the study. HIV counseling was provided to families of participating children undergoing HIV testing.

## 3. Results

### 3.1 Study population characteristics

A total of 576 pneumonia cases were recruited. Of these, 472 had a plasma sample for biomarker measurement and were included in this analysis. In 11 out of 472 pneumonia cases, CRP could not be assessed due to insufficient volume for all assays. Their clinical and demographic characteristics are presented in Table 1. Half (50.0%; 236/472) were females, median age was 14.1 months (interquartile range [IQR]: 6.1 to 27.1), 17.6% (83/472) had severe malnutrition (weight-for-age z-score (WAZ) <-3 SD), 18.6% (88/472) were HIV positive, 18.6% (88/472) had malaria, 13.1% (62/472) had an invasive bacterial disease, 65.1% (298/458) had a viral respiratory infection, and 37.4% (151/404) had a normal chest X-ray. A total of 15 (3.2%) died during hospital stay, 20 (4.2%) absconded, 20 (4.2%) were transferred to another hospital, and 417 (88.3%) were discharged alive.

**Table 1:** Demographic and clinical characteristics of the study population.

**Variable** ^**a**^	**Healthy community controls (n = 80)**	**All pneumonia cases (n = 472)**	**Pneumonia cases**			
			**Discharged alive (n = 417)**	**In-hospital deaths (n = 15)**	**Transferred (n = 20)**	**Absconders (n = 20)**
**Female sex**	45 (56.3)	236 (50.0)	205 (49.2)	6 (40.0)	14 (70.0)	11 (55.0)
**Age**						
<2 months	1 (1.3)	27 (5.7)	22 (5.3)	1 (6.7)	2 (10.0)	2 (10.0)
2 to 11 months	8 (10.0)	178 (37.7)	155 (37.2)	7 (46.7)	7 (35.0)	9 (45.0)
12 to 59 months	59 (73.8)	231 (48.9)	209 (50.1)	5 (33.3)	8 (40.0)	9 (45.0)
5 to <10 years	12 (15.0)	36 (7.6)	31 (7.4)	2 (13.3)	3 (15.0)	0 (0)
Median (months)	39.0 [24.7, 55.8]	14.1 [6.1, 27.1]	14.9 [6.4, 27.7]	10.5 [3.0, 24.1]	13.8 [5.4, 19.8]	7.9 [4.3, 20.4]
**WAZ** ^**b**^						
>-1	46/68 (67.6)	176 (37.3)	159 (38.1)	1 (6.7)	6 (30.0)	10 (50.0)
-1 to -3	22/68 (32.4)	213 (45.1)	192 (46.0)	6 (40.0)	9 (45.0)	6 (30.0)
<-3	0/68 (0)	83 (17.6)	66 (15.8)	8 (53.3)	5 (25.0)	4 (20.0)
Median	-0.6 [-1.3, 0.0], n = 68	-1.5 [-2.7, -0.5]	-1.5 [-2.5, -0.4]	-3.4 [-4.2, -1.6]	-1.7 [-3.2, -0.8]	-1.0 [-2.8, -0.4]
**MUAC (cm)**	15.0 [14.0, 16.0], n = 68	14.0 [13.0, 15.0], n = 464	14.0 [13.0, 15.0], n = 409	12.0 [10.0, 13.0]	13.0 [11.5, 15.0]	13.0 [12.0, 14.5]
**HIV positive status**	-	88 (18.6)	72 (17.3)	7 (46.7)	6 (30.0)	3 (15.0)
**Malaria parasitemia**	-	88 (18.6)	81 (19.4)	1 (6.7)	3 (15.0)	3 (15.0)
**Invasive bacterial disease** ^**c**^	-	62 (13.1)	45 (10.8)	7 (46.7)	6 (30.0)	4 (20.0)
**Viral respiratory infection** ^**d**^	-	298/458 (65.1)	269/405 (66.4)	7/14 (50.0)	10 (50.0)	12/19 (63.2)
**Axillary temperature**	-	38.1 [37.1, 39.1]	38.1 [37.1, 39.1]	37.6 [36.5, 38.7]	37.9 [37.2, 39.0]	37.6 [37.0, 38.4]
**Fever (≥37.5°C, axillary temperature)**	-	323 (68.4)	290 (69.5)	8 (53.3)	14 (70.0)	11 (55.0)
**SpO**_**2**_ **(%)**	-	97 [96, 98], n = 448	97 [96, 98], n = 397	97 [91, 97]	96 [94, 97], n = 17	97 [96, 98], n = 19
**Hypoxemia (SpO**_**2**_ **<90%)**	-	11/448 (2.5)	8/397 (2.0)	1 (6.7)	1/17 (5.9)	1/19 (5.3)
**Cyanosis**	-	16 (3.4)	14 (3.4)	1 (6.7)	1 (5.0)	0 (0)
**Respiratory rate**	-	56 [50, 63]	56 [49, 62]	54 [44, 60]	58 [49, 69]	58.0 [52, 66]
**Lower chest wall indrawing**	-	315 (66.7)	278 (66.7)	11 (73.3)	12 (60.0)	14 (70.0)
**Nasal flaring**	-	226 (47.9)	198 (47.5)	7 (46.7)	8 (40.0)	13 (65.0)
**Deep breathing**	-	54 (11.4)	44 (10.6)	3 (20.0)	2 (10.0)	5 (25.0)
**Grunting**	-	54 (11.4)	45 (10.8)	3 (20.0)	3 (15.0)	3 (15.0)
**Wheezing**	-	105 (22.3)	99 (23.7)	2 (13.3)	0 (0)	4 (20.0)
**Crackles**	-	261 (55.3)	232 (55.6)	10 (66.7)	7 (35.0)	12 (60.0)
**Ronchi**	-	162 (34.3)	147 (35.3)	4 (26.7)	3 (15.0)	8 (40.0)
**Inspiratory stridor**	-	20 (4.2)	18 (4.3)	0 (0)	0 (0)	2 (10.0)
**Altered consciousness (BCS<5)**	-	18 (3.8)	15 (3.6)	1 (6.7)	1 (5.0)	1 (5.0)
**Coma (BCS<3)**	-	3 (0.6)	1 (0.24)	0 (0)	1 (5.0)	1 (5.0)
**Prostration**	-	44 (9.3)	35 (8.4)	2 (13.3)	2 (10.0)	5 (25.0)
**Convulsions**	-	26/462 (5.6)	24/408 (5.9)	1 (6.7)	1 (5.0)	0/19 (0)
**Unable to drink/breastfeed**	-	22/461 (4.8)	18/407 (4.4)	2 (13.3)	1 (5.0)	1/19 (5.3)
**Dehydration**	-	29/462 (6.3)	22/408 (5.4)	5 (33.3)	0 (0)	2/19 (10.5)
**Radiological findings**						
Normal	-	151/404 (37.4)	136/367 (37.1)	2/8 (25.0)	5/13 (38.5)	8/16 (50.0)
Other infiltrates	-	89/404 (22.0)	83/367 (22.6)	2/8 (25.0)	2/13 (15.4)	2/16 (12.5)
Endpoint pneumonia	-	164/404 (40.6)	148/367 (40.3)	4/8 (50.0)	6/13 (46.2)	6/16 (37.5)
**Glucose (mmol/L)**	-	6.0 [5.3, 7.0], n = 424	6.1 [5.3, 7.0], n = 377	5.9 [4.3, 7.7], n = 13	5.9 [5.4, 6.0], n = 18	5.8 [5.4, 7.0], n = 16
**Hematocrit (%)**	-	27.4 [22.9, 31.1], n = 470	27.5 [23.1, 31.3], n = 415	24.3 [22.9, 26.8]	24.4 [19.6, 30.2]	28.6 [23.2, 31.2]
**WBC count (10** ^**9**^ **/L)**	-	14.3 [10.1, 20.4], n = 448	14.2 [10.1, 20.4], n = 394	14.6 [12.2, 21.1]	11.8 [9.7, 17.1]	15.4 [12.9, 22.0], n = 19
**WHO severity definition**	-	220 (46.6)	193 (46.3)	10 (66.7)	7 (35.0)	10 (50.0)
**LODS**	-	0 [0, 0]	0 [0, 0]	0 [0, 1]	0 [0, 0]	0 [0, 1]
**RISC-Malawi score**	-	1 [0, 4], n = 441	1 [0, 4], n = 390	5 [2, 8]	4 [1, 8], n = 17	3 [1, 8], n = 19
**Length of hospital stay (days)**	-	3.9 [2.8, 5.8]	3.9 [2.9, 5.4]	3.1 [0.9, 6.8]	2.5 [1.1, 6.4]	4.5 [2.5, 5.9]
**Antibiotic therapy**	-	415/460 (90.2)	369/408 (90.4)	13/14 (92.9)	17 (85.0)	16/18 (88.9)
**Antimalarial therapy**	-	108/459 (23.5)	97/407 (23.8)	2/14 (14.3)	4 (20.0)	5/18 (27.8)
**Previous admissions for pneumonia**	-	16 (3.4)	12 (2.9)	3 (20.0)	0 (0)	1 (5.0)

^a^ Data presented as median [interquartile range] or frequency (percent) as appropriate. Number of subjects indicated if presence of missing values.

^b^ Calculated using the LMS method and the 2000 US CDC Growth Reference data [40].

^c^ Defined as the detection of a non-contaminating bacterial organism in blood, cerebrospinal fluid, and/or pleural fluid through culture or PCR analysis of dried blood spots for *Streptococcus pneumoniae* and *Haemophilus influenzae* type B.

^d^ At least one virus detected in nasopharyngeal aspirate. Virus targeted included: adenovirus, bocavirus, coronavirus (229E and OC43), enterovirus, influenza virus (A, B, and C), metapneumovirus, parainfluenza virus (1, 2, 3, and 4), respiratory syncytial virus (A and B), and rhinovirus.

Abbreviations: BCS (Blantyre Coma Scale), HIV (human immunodeficiency virus), LODS (Lambaréné Organ Dysfunction Score), MUAC (mid-upper arm circumference), RISC-Malawi (Respiratory Index of Severity in Children-Malawi), WAZ (weight-for-age z-score), WBC (white blood cell), WHO (World Health Organization).

Of 117 community controls examined, 80 fulfilled the definition of healthy community control and had a plasma sample for biomarker measurement. Of these, 56.3% (45/80) were female and median age was 39.0 months (IQR: 24.7 to 55.8) (Table 1).

### 3.2 Biomarkers as indicators of disease and severity

**Fig 1:**
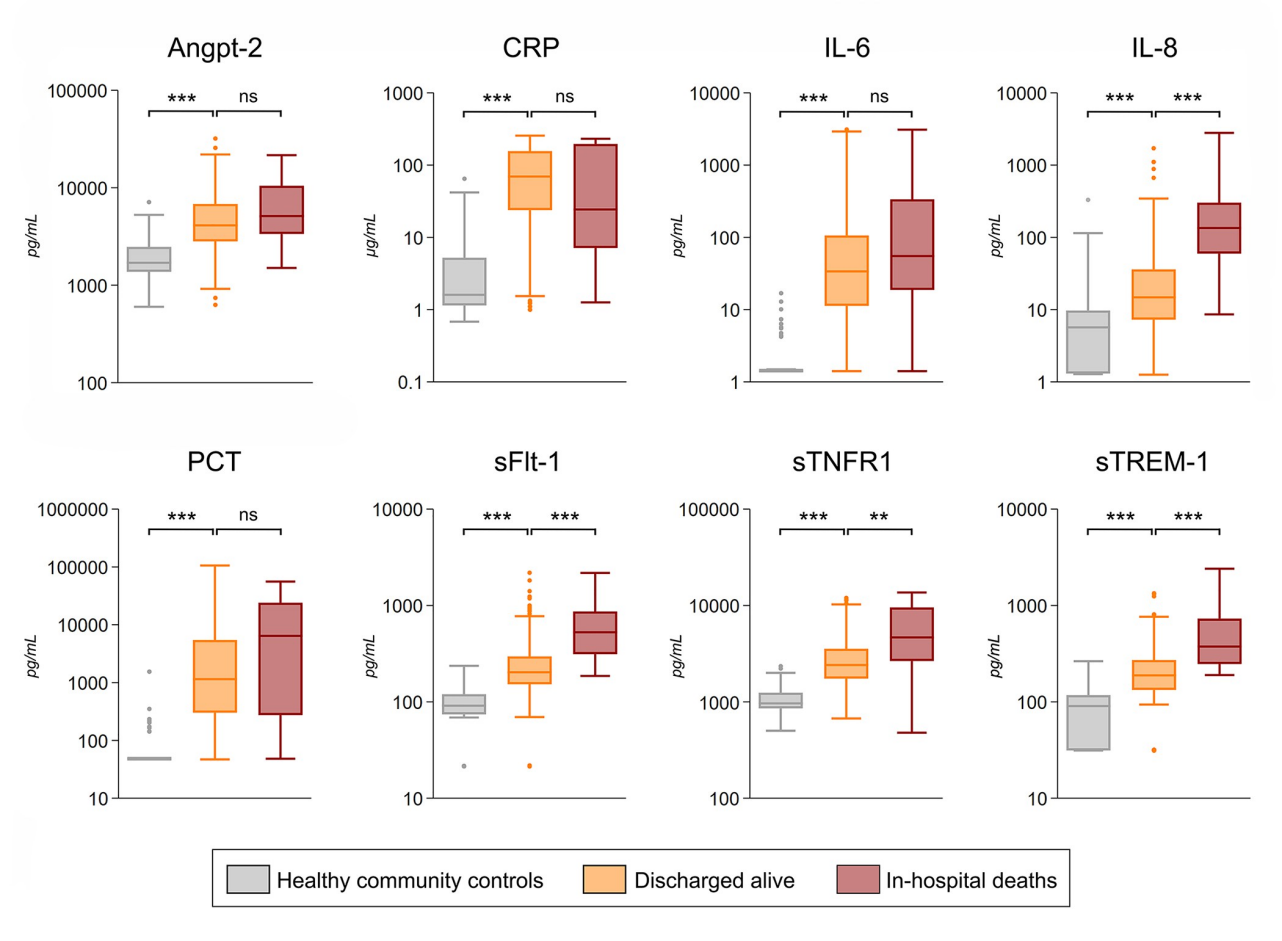
Plasma concentrations of immune and endothelial activation biomarkers at study enrolment in healthy community controls, pneumonia cases discharged alive, and pneumonia cases who died during hospital stay. Boxplots with median and interquartile range. Whiskers represent one and a half times the interquartile range, and outliers are plotted as individual points. Concentrations are in pg/mL, except CRP in μg/mL. p-values were computed using the Mann-Whitney U test. *p<0.05, **<0.01; ***p<0.001; ns: non-significant. Abbreviations: Angpt-2 (angiopoietin-2), CRP (C-reactive protein), IL-6 (interleukin-6), IL-8 (interleukin-8), PCT (procalcitonin), sFlt-1 (soluble fms-like tyrosine kinase-1), sTNFR1 (soluble tumor necrosis factor receptor), sTREM-1 (soluble triggering receptor expressed on myeloid cells 1).

All biomarker concentrations were significantly higher in pneumonia cases versus healthy community controls (p<0.001). Equivalent results were observed when comparing pneumonia cases discharged alive with healthy community controls (Fig 1).

Forty-six percent of pneumonia cases fulfilled the WHO definition of severe pneumonia. IL-8 was the only biomarker significantly increased in severe cases compared to non-severe cases (p = 0.046) (S2 Table).

### 3.3 Biomarkers as indicators of in-hospital death

There were 15 in-hospital deaths in children with pneumonia during initial hospital admission. These occurred between the day of admission and 16 days afterwards, with 40.0% (6/15) happening within the first 48 hours of hospitalization. IL-8, sFlt-1, and sTREM-1 were higher in in-hospital deaths compared to children discharged alive (p<0.001), as well as sTNFR1 (p = 0.002) (Fig 1). The odds of in-hospital mortality increased significantly for a two-fold increase in IL-8, sFlt-1, sTREM-1, or sTNFR1 concentration (Table 2).

**Table 2:** Associations of biomarkers with primary and secondary outcomes using univariable logistic regression.

	In-hospital mortality		28-day mortality^a^		90-day mortality^a^	
**Biomarker**	**OR (95% CI)** ^**b**^	**p-value**	**OR (95% CI)** ^**b**^	**p-value**	**OR (95% CI)** ^**b**^	**p-value**
Angpt-2	1.51 (0.88, 2.62)	0.137	1.60 (1.01, 2.55)	0.047	1.48 (0.97, 2.23)	0.066
CRP	0.82 (0.64, 1.04)	0.102	0.82 (0.68, 1.01)	0.059	0.79 (0.66, 0.95)	0.010
IL-6	1.15 (0.94, 1.40)	0.189	1.12 (0.94, 1.32)	0.209	1.06 (0.91, 1.24)	0.428
IL-8	2.05 (1.56, 2.69)	<0.001	1.86 (1.47, 2.35)	<0.001	1.76 (1.42, 2.17)	<0.001
PCT	1.11 (0.94, 1.32)	0.229	1.07 (0.93, 1.23)	0.337	1.02 (0.90, 1.15)	0.785
sFlt-1	3.55 (2.13, 5.91)	<0.001	3.40 (2.12, 5.45)	<0.001	2.62 (1.73, 3.96)	<0.001
sTNFR1	3.11 (1.68, 5.73)	<0.001	2.52 (1.50, 4.22)	<0.001	2.03 (1.28, 3.22)	0.003
sTREM-1	3.66 (2.10, 6.37)	<0.001	2.88 (1.77, 4.67)	<0.001	2.63 (1.69, 4.08)	<0.001

^a^ Combining both in-hospital and post-discharge deaths after initial admission.

^b^ ORs, 95% CIs, and p-values are from univariable logistic regression models. ORs presented indicate the increase in odds of the outcome for a two-fold increase in each biomarker concentration.

Abbreviations: Angpt-2 (angiopoietin-2), CI (confidence interval), CRP (C-reactive protein), IL-6 (interleukin-6), IL-8 (interleukin-8), OR (odds ratio), PCT (procalcitonin), sFlt-1 (soluble fms-like tyrosine kinase-1), sTNFR1 (soluble tumor necrosis factor receptor), sTREM-1 (soluble triggering receptor expressed on myeloid cells 1).

**Fig 2:**
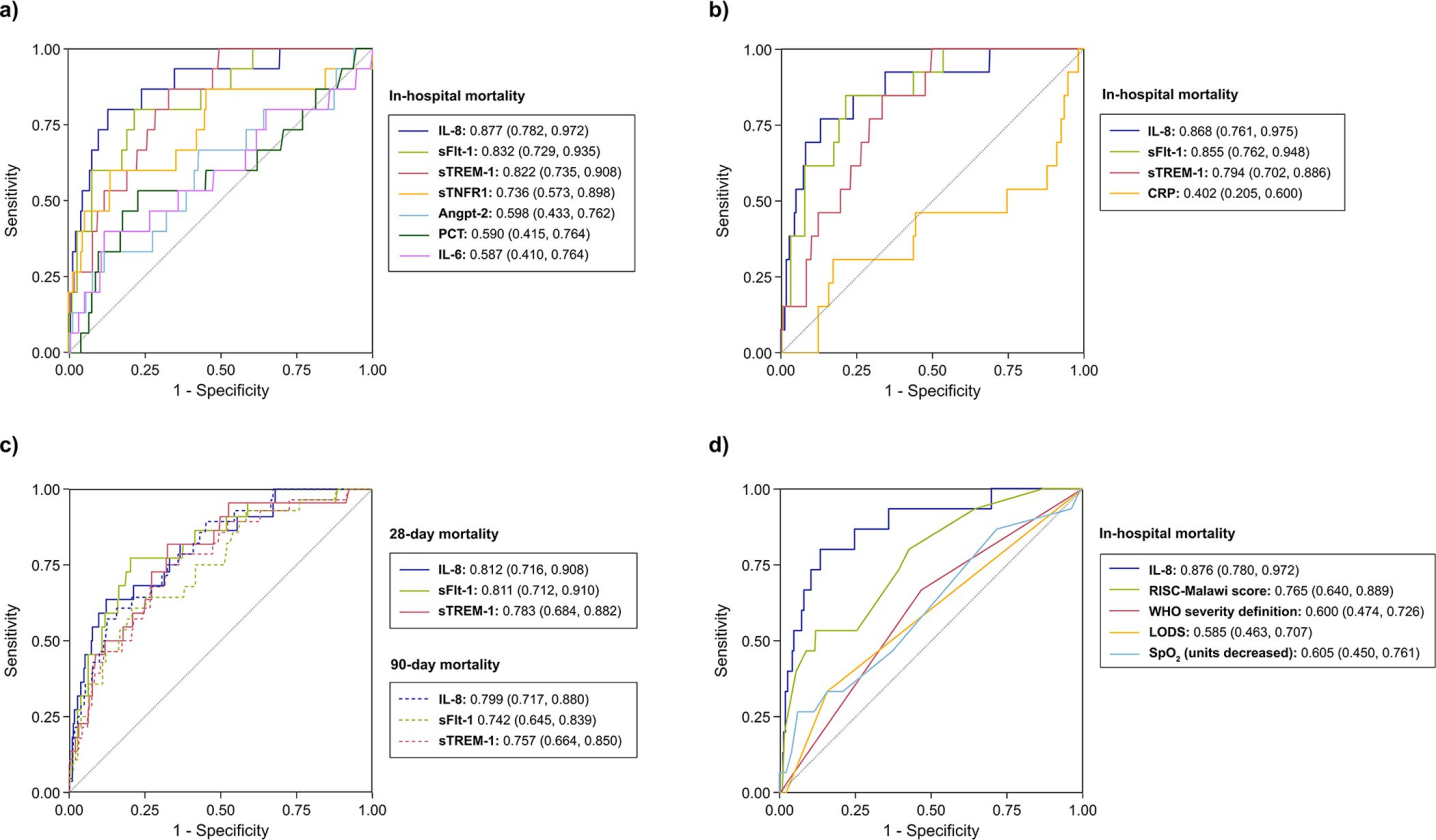
AUROCs of immune and endothelial activation biomarkers and clinical parameters. (A) IL-8, sFlt-1, sTREM-1, sTNFR1, Angpt-2, PCT, and IL-6 AUROCs for in-hospital deaths (n = 432). (B) IL-8, sFlt-1, sTREM-1, and CRP AUROCs for in-hospital deaths (n = 421). (C) IL-8, sFlt-1, and sTREM-1 AUROCs for 28-day and 90-day mortality (in-hospital & post-discharge) (n = 309). (D) IL-8 and clinical parameters AUROCs for in-hospital deaths (n = 405). Abbreviations: Angpt-2 (angiopoietin-2), AUROC (area under the receiver operating characteristic curve), CRP (C-reactive protein), IL-6 (interleukin-6), IL-8 (interleukin-8), LODS (Lambaréné Organ Dysfunction Score), PCT (procalcitonin), RISC-Malawi (Respiratory Index of Severity in Children-Malawi), sFlt-1 (soluble fms-like tyrosine kinase-1), sTNFR1 (soluble tumor necrosis factor receptor), sTREM-1 (soluble triggering receptor expressed on myeloid cells 1), WHO (World Health Organization).

IL-8 had the best discriminatory capacity for in-hospital deaths, with an AUROC of 0.877 (95% confidence interval [CI]: 0.782 to 0.972) (Fig 2A and 2B). However, IL-8 was not significantly better than the next two most discriminative biomarkers, sFlt-1 and sTREM-1, with AUROCs of 0.832 (95% CI: 0.729 to 0.935) and 0.822 (95% CI: 0.735 to 0.908), respectively. These three biomarkers individually were significantly superior to CRP and PCT (S3 Table). IL-8 combined with sTREM-1 was the best two-biomarker combination (AUROC: 0.904 [95% CI: 0.820 to 0.988]), which performed slightly better than IL-8 alone (p = 0.024). Correlations between biomarkers can be found in S1 Fig.

### 3.4 Biomarkers as indicators of 28-day and 90-day mortality

HDSS data provided long-term vital status information for 65.5% (309/472) of pneumonia cases. There were 22 deaths within 28 days of hospital admission, and 28 deaths within 90 days. These correspond to the 15 children dying during the initial hospital admission and 7 or 13 children dying after hospital discharge. Deaths happening after hospital discharge were from children either discharged alive (10/13) or transferred to another hospital (3/13). As per HDSS data, 5/13 post-discharge deaths occurred at home, 3/13 in a health facility, and 5/13 in an unspecified location.

IL-8, sFlt-1, sTREM-1 (p<0.001) and sTNFR1 (p = 0.004) were significantly elevated in children who subsequently died at 28 days. IL-8, sFlt-1, and sTREM-1 remained most significantly elevated in those dying at 90 days (p<0.001), as was sTNFR1 (p = 0.011) (S4 Table). ORs are provided in Table 2. IL-8, sFlt-1, and sTREM-1 were the best-performing biomarkers for discriminating deaths at 28 or 90 days, with AUROCs ranging from 0.742 (95% CI: 0.645 to 0.839) to 0.812 (95% CI: 0.716 to 0.908) (Fig 2C, S3 Table). Best two-biomarker combination for 28-day mortality was IL-8 and sFlt-1 (AUROC: 0.847 [95% CI: 0.770 to 0.924]), while for 90-day mortality was IL-8 and sTREM-1 (AUROC: 0.828 [95% CI: 0.742 to 0.914]). However, two-biomarker models did not outperform models with only IL-8 (p≥0.05).

The sensitivity analysis evaluating secondary outcomes only for children not dying during initial hospital admission showed similar results. Best AUROCs for 28-day mortality also were from sFlt-1 (0.744 [95% CI: 0.523 to 0.965]), sTREM-1 (0.686 [95% CI: 0.447 to 0.925]), and IL-8 (0.659 [95% CI: 0.473 to 0.845]); and for 90-day mortality the best ones were from IL-8 (0.700 [95% CI: 0.584 to 0.816]), sTREM-1 (0.672 [95% CI: 0.516 to 0.833]), and sFlt-1 (0.628 [95% CI: 0.478 to 0.777]) (S5 Table).

### 3.5 Biomarkers compared to clinical parameters to predict in-hospital death

Of the four clinical variables selected *a priori*, the RISC-Malawi score (OR: 1.38 [95% CI: 1.18 to 1.61], p<0.001) and SpO_2_ (OR: 1.19 [95% CI: 1.02 to 1.41] per unit decreased, p = 0.049) were associated with in-hospital mortality in univariable analysis (S6 Table). IL-8, sFlt-1, and sTREM-1 ORs for in-hospital mortality remained similar when adjusting for one of the pre-specified clinical variables at a time.

IL-8 performed significantly better in discriminating in-hospital mortality compared to the WHO severity definition, LODS, and SpO_2_ (p<0.001) (Fig 2D). sFlt-1 and sTREM-1 also performed significantly better than these three clinical parameters (p<0.009). IL-8, sFlt-1, and sTREM-1 were better than the RISC-Malawi score but differences were non-significant (p = 0.087, p = 0.466, and p = 0.429, respectively). When adding IL-8, sFlt-1, or sTREM-1 to a model with one of these clinical parameters, AUROCs improved on all occasions (non-significantly for the addition of sTREM-1 to RISC-Malawi score) (Table 3). Of note, models with one of these three biomarkers alone performed as well as models combining the same biomarker with SpO_2_ or a severity score/definition (p>0.05).

**Table 3:** AUROCs to discriminate between children discharged alive and in-hospital deaths for clinical variables alone or in combination with best-performing biomarkers.

Variables (n = 405)	AUROC (95% CI)	+ IL-8, AUROC (95% CI)	p-value^a^	+ sFlt-1, AUROC (95% CI)	p-value^a^	+ sTREM-1, AUROC (95% CI)	p-value^a^
RISC-Malawi score	0.765 (0.640, 0.889)	0.886 (0.790, 0.982)	0.023	0.896 (0.830, 0.962)	0.019	0.845 (0.754, 0.936)	0.062
SpO_2_ (units decreased)	0.605 (0.450, 0.761)	0.861 (0.754, 0.968)	<0.001	0.829 (0.721, 0.937)	<0.001	0.817 (0.723, 0.911)	0.004
WHO severity definition	0.600 (0.474, 0.726)	0.875 (0.774, 0.976)	<0.001	0.826 (0.716, 0.936)	<0.001	0.832 (0.731, 0.932)	<0.001
LODS	0.585 (0.463, 0.707)	0.876 (0.780, 0.972)	<0.001	0.836 (0.740, 0.932)	<0.001	0.816 (0.722, 0.909)	<0.001
IL-8	0.876 (0.780, 0.972)						
sFlt-1	0.830 (0.727, 0.933)						
sTREM-1	0.817 (0.730, 0.904)						

^a^ AUROCs comparison of the models with a clinical parameter alone versus the models with the same clinical parameter combined with either IL-8, sFlt-1, or sTREM-1. p-values were computed using the algorithm suggested by DeLong *et al*. [34].

Abbreviations: AUROC (area under the receiver operating characteristic curve), CI (confidence interval), IL-8 (interleukin-8), LODS (Lambaréné Organ Dysfunction Score), RISC-Malawi (Respiratory Index of Severity in Children-Malawi), sFlt-1 (soluble fms-like tyrosine kinase-1), sTREM-1 (soluble triggering receptor expressed on myeloid cells 1), WHO (World Health Organization).

### 3.6 Using biomarkers for risk-stratification

Cut-offs for in-hospital mortality maximizing sensitivity and specificity (Youden’s index method) are reported in S7 Table. IL-8 was the biomarker with a cut-off associated with best performance estimators, as a 59.6 pg/mL threshold gave a sensitivity of 80.0 (95% CI: 51.9 to 95.7) and a specificity of 86.8 (95% CI: 83.2 to 89.9).

We also used a pre-specified LR+ of 10 and a LR- of 0.1 to define cut-offs and place children with pneumonia into low, intermediate and high-risk groups. We could only obtain these cut-offs for IL-8, sFlt-1, and sTREM-1, as other biomarkers were not good enough. Table 4 shows these values and their corresponding performance estimators. When we applied the cut-offs of <239 pg/mL and ≥629 pg/mL for sTREM-1 based on Leligdowicz *et al*. [13], this resulted in 1.0% (3/290) in-hospital deaths occurring among children placed in the low-risk group, 6.4% (8/125) among those in the intermediate-risk group, and 23.5% (4/17) among those in the high-risk group (Table 4).

**Table 4:** Biomarker cut-offs for in-hospital mortality and performance estimators.

Biomarker	Cut-off (pg/mL)	LR+(95% CI)	LR-(95% CI)	Sensitivity (95% CI)	Specificity (95% CI)	PPV (95% CI)	NPV (95% CI)	In-hospital deaths within each risk group		
								Low-risk (%)	Intermediate-risk (%)	High-risk (%)
IL-8^a^	<25.4	2.67 (2.21, 3.22)	0.10 (0.02, 0.68)	93.3 (68.1, 99.8)	65.0 (60.2, 69.6)	8.8 (4.9, 14.2)	99.6 (98.0, 100.0)	1/272 (0.4)	6/130 (4.6)	8/30 (26.7)
	≥124.5	10.11 (5.42, 18.87)	0.49 (0.29, 0.85)	53.3 (26.6, 78.7)	94.7 (92.1, 96.7)	26.7 (12.3, 45.9)	98.3 (96.4, 99.3)			
sFlt-1^a^	<197.7	1.75 (1.48, 2.05)	0.14 (0.02, 0.95)	93.3 (68.1, 99.8)	46.5 (41.7, 51.4)	5.9 (3.3, 9.7)	99.5 (97.2, 100.0)	1/195 (0.5)	8/215 (3.7)	6/22 (27.3)
	≥745.2	10.43 (4.76, 22.84)	0.62 (0.41, 0.94)	40.0 (16.3, 67.7)	96.2 (93.8, 97.8)	27.3 (10.7, 50.2)	97.8 (95.9, 99.0)			
sTREM-1^a^	<197.6	1.97 (1.66, 2.33)	0.13 (0.02, 0.85)	93.3 (68.1, 99.8)	52.5 (47.6, 57.4)	6.6 (3.7, 10.8)	99.5 (97.5, 100.0)	1/220 (0.5)	10/197 (5.1)	4/15 (26.7)
	≥670.8	10.11 (3.64, 28.09)	0.75 (0.55, 1.02)	26.7 (7.8, 55.1)	97.4 (95.3, 98.7)	26.7 (7.8, 55.1)	97.4 (95.3, 98.7)			
sTREM-1^b^	<239.0	2.57 (1.92, 3.43)	0.29 (0.11, 0.80)	80.0 (51.9, 95.7)	68.8 (64.1, 73.2)	8.5 (4.4, 14.3)	99.0 (97.0, 99.8)	3/290 (1.0)	8/125 (6.4)	4/17 (23.5)
	≥629.0	8.55 (3.16, 23.14)	0.76 (0.56, 1.03)	26.7 (7.8, 55.1)	96.9 (94.7, 98.3)	23.5 (6.8, 49.9)	97.3 (95.3, 98.7)			

^a^ Cut-offs derived from our dataset and associated with a LR+ of 10 or a LR- of 0.1.

^b^ Cut-offs applied from Leligdowicz *et al*. [13].

Abbreviations: CI (confidence interval), IL-8 (interleukin-8), LR+ (positive likelihood ratio), LR- (negative likelihood ratio), NPV (negative predictive value), PPV (positive predictive value), sFlt-1 (soluble fms-like tyrosine kinase-1), sTREM-1 (soluble triggering receptor expressed on myeloid cells 1).

## 4. Discussion

In this cohort of hospitalized Mozambican children fulfilling WHO diagnostic criteria for pneumonia, IL-8, sFlt-1, and sTREM-1 concentration determined at hospital admission showed good individual prognostic accuracy for in-hospital and longer-term mortality. They outperformed other commonly used clinical parameters and were unaltered in healthy community controls.

IL-8 had the highest discriminatory capacity for severity and in-hospital deaths. IL-8 is a chemokine produced by different blood cells upon stimulation with inflammatory stimuli, which attracts and activates neutrophils and other inflammatory cells. sFlt-1 and sTREM-1 were the next best prognostic biomarkers for in-hospital mortality. sFlt-1 regulates angiogenesis and inflammation-induced vascular permeability, and sTREM-1 is thought to contribute to immunosuppression via inhibition and apoptosis of neutrophils and monocytes. In accordance with our results, a previous study in 2,502 hospitalized Ugandan febrile children reported sTREM-1, followed by sFlt-1 and IL-8, as best prognostic biomarkers for 7-day in-hospital mortality [13]. When limiting the cohort to those children who met the IMCI diagnostic criteria for pneumonia, these three biomarkers remained the best ones for 48-hour in-hospital mortality [21]. Another study in Bhutanese children hospitalized with clinical pneumonia showed that sTREM-1 was the best prognostic biomarker for a composite outcome of poor prognosis [41]. In a study in India, IL-8 levels were also higher in fatal cases [42]. Moreover, IL-8 and sTREM-1 have been reported to be increased in severe childhood pneumonia cases [11, 43].

In this study, CRP and PCT were significantly elevated in pneumonia cases compared to controls, but they were neither good severity indicators nor prognosticators of death. A systematic review and meta-analysis found them to be significantly increased in children with severe pneumonia compared to those with non-severe pneumonia [11]. However, almost all included studies were conducted in high-income countries, and PCT and CRP have been suggested to be influenced by HIV, malaria, and malnutrition [14, 44, 45]. Other reviews reported limited or conflicting results [35, 46]. Studies conducted in sub-Saharan Africa have shown that they are poor indicators of childhood pneumonia severity and outcome [15–21], including a previous study in Manhiça [14].

Studies in children have found that biomarkers of immune and endothelial activation are better prognostic indicators than common clinical markers of pneumonia disease severity [21, 41]. Hypoxemia is considered a hallmark of pneumonia severity [35], but SpO_2_ had poor discriminating performance for in-hospital mortality in our study cohort (with an exclusion criterion of SpO_2_ ≤85%). The severity dichotomization based on the WHO 2013 pocket book [3] did not have good prognostic accuracy either. In fact, we did not find clear biomarker differences between these severity groups. Nonetheless, it is important to note that we applied this definition to children older than five years old. RISC-Malawi [7, 36] and LODS [37–39] were selected as relevant scores developed with data from African children to predict in-hospital mortality. The RISC-Malawi score demonstrated the best discrimination for in-hospital deaths among pre-specific clinical parameters and was the only one not significantly different from best-performing biomarkers. Remarkably, the addition of IL-8, sFlt-1, or sTREM-1 to a model with one of these clinical parameters improved AUROCs. Hyperlactatemia has been identified as a promising prognostic indicator in children with pneumonia [47, 48], although inferior to sTREM-1 [21]. Unfortunately, we lacked lactate data, and further studies are needed to evaluate its prognostic accuracy in comparison to the studied biomarkers.

Children at high risk of adverse outcomes could be prioritized for resources and higher level of care as they are more likely to benefit, while those at low risk could be confidently discharged and iatrogenic harm prevented [12, 49]. Biomarker cut-offs were derived and tested to exemplify biomarker potential, but a larger sample size is needed to provide definitive values. Moreover, cut-offs would need to be adapted to the specific use-case and consider both benefits and costs based on decision theory. Interestingly, when calculating different biomarker cut-offs, optimal cut-offs of sTREM-1 based on pre-specified likelihood ratios were similar to those derived by Leligdowicz *et al*. in a cohort of hospitalized febrile children [13]. The potential of these biomarkers is not limited to pediatric pneumonia, but could be used in a myriad of situations where children are at risk of severe and fatal infections. In fact, in this study we included children with WHO-defined clinical pneumonia, a heterogeneous syndromic group in which we detected diverse pathogens (bacteria, viruses, and malaria parasites). Our study population does not include SARS-CoV-2 infections, but recent studies have also highlighted the utility of IL-8, sFlt-1, and sTREM-1 to risk-stratify adult patients with COVID-19 pneumonia [50–53].

IL-8, sFlt-1, and sTREM-1 had also the best predicting value for 28-day and 90-day mortality after initial hospital admission. When limiting analyses to deaths occurring after hospital discharge (sensitivity analysis), we obtained the same best-performing biomarkers. As in similar settings, the burden of post-discharge mortality in Manhiça is not trivial [54, 55]. Children discharged after treatment for severe pneumonia are at increased risk of relapse, morbidities and mortality [56, 57], but adequate follow-up of all children is often unfeasible and unaffordable in low-income settings. Host biomarkers could improve discharge follow-up by enabling the early identification of children at higher risk of post-discharge complications, therefore improving survival after hospitalization [12, 49].

Measuring a panel of biomarkers from different pathophysiological pathways could improve outcome prognostication compared to measuring individual biomarkers [58]. However, two-biomarker combinational approaches did not importantly increase mortality discrimination compared to a more parsimonious approach with only IL-8. Although the statistical power of this analysis is limited due to the low numbers of events in this cohort, these results are in agreement with previous studies evaluating similar biomarkers in children with pneumonia or febrile adult populations, where one biomarker provided comparable mortality discrimination to few biomarkers combined [21, 59, 60].

This study benefits from a prospective design and exhaustive clinical data collection. Major strengths were the addition of a healthy community control group and having post-discharge mortality data. Nevertheless, the number of deaths limited our statistical power. For this reason, we constructed models with only two independent variables, pre-specified few relevant clinical parameters for comparison with biomarkers, did not attempt to derive a full risk prediction model nor validate one, and did not explore if prognostic performance interacted with other factors (e.g., HIV status, malnutrition, age, or pathogens detected). This limitation, common in other studies, highlights the need for individual patient meta-analyses. Besides, HDSS mortality data were not specifically collected for the purpose of this study and this could have impacted the accuracy of the dates of post-discharge deaths.

In this study, we provide evidence that IL-8, sFlt-1, and sTREM-1 are promising candidates to be used in the risk-stratification of childhood pneumonia. Nonetheless, further studies are necessary to establish with assurance which biomarkers and cut-offs are most useful in different use-cases and in combination with clinical parameters. Another important step is the incorporation of these promising biomarkers into rapid, easy-to-use, and inexpensive point-of-care triage tests to be used in low-resource settings. Their hypothetical implementation into clinical practice could transform risk-stratification and contribute in efforts to reduce pneumonia mortality among the most vulnerable children.

## Supporting information

S1 Table Luminex and ELISA biomarker dynamic ranges and distribution of values outside the dynamic range. (DOCX)

S2 Table Biomarker concentrations in pneumonia cases by severity group. (DOCX)

S3 Table Biomarker AUROCs for primary and secondary study outcomes. (DOCX)

S4 Table Biomarker concentrations in 28-day or 90-day deaths and survivors. (DOCX)

S5 Table Sensitivity analysis: Biomarker AUROCs for 28-day or 90-day deaths occurring only at post-discharge. (DOCX)

S6 Table Demographic and clinical characteristics associated with in-hospital mortality in pneumonia cases. (DOCX)

S7 Table Biomarker performance estimators to identify in-hospital mortality using cut-offs based on the Younden index. (DOCX)

S1 Fig Biomarker Spearman’s correlation coefficients in pneumonia cases. (DOCX)
