# Supplementary material for: Prognostic accuracy of biomarkers of immune and endothelial activation in Mozambican children hospitalized with pneumonia
Source: PLOS Glob Public Health. 2023 Feb 23;3(2):e0001553. doi: 10.1371/journal.pgph.0001553 (PMC10021812; doi:10.1371/journal.pgph.0001553)
Supplement: S1 Table — (DOCX) [file pgph.0001553.s001.docx]

**S1 Table. Luminex and ELISA biomarker dynamic ranges and distribution of values outside the dynamic range**

| **Biomarker** | **All study participants (n=552)** | | **Healthy controls (n=80)** | | **Pneumonia cases (n=472)** | |
| --- | --- | --- | --- | --- | --- | --- |
|  | **Dynamic range (pg/mL)** | **Percentage of samples outside the dynamic range (n/N)** | **Number of samples below detection** | **Number of samples above detection** | **Number of samples below detection** | **Number of samples above detection** |
| **Luminex^a^:** | | | | | | |
| Angpt-2 | 22.5 −16430 | 0 (0/552) | 0 | 0 | 0 | 0 |
| IL-6 | 1.4 − 1030 | 18.5 (102/552) | 68 | 0 | 27 | 7 |
| IL-8 | 1.3 − 930 | 13.0 (72/552) | 23 | 0 | 47 | 2 |
| PCT | 2.9 − 2110 | 23.2 (128/552) | 71 | 0 | 55 | 2 |
| sFlt-1 | 21.4 − 15610 | 3.3 (18/552) | 13 | 0 | 5 | 0 |
| sTNFR1 | 19.4 − 14120 | 0 (0/552) | 0 | 0 | 0 | 0 |
| sTREM-1 | 31.4 − 22870 | 13.4 (74/552) | 39 | 0 | 35 | 0 |
| **ELISA^b^:** | | | | | | |
| CRP | 15.6 − 1000 | 22.4 (121/541^c^) | 55 | 0 | 28 | 38 |

^a^ Biomarkers were processed using two separate Luminex panels. Panel 1 was performed at a 1:3 dilution and included IL-6, IL-8, sFlt-1, sTNFR1, and sTREM-1. Panel 2 was performed at a 1:50 dilution and only included PCT.

^b^ Plasma samples were diluted at 1:250,000 for CRP.

^c^ 11 samples had insufficient volume for both assays, and Luminex was prioritized over ELISA.

Abbreviations: Angpt-2 (angiopoietin-2), CRP (C-reactive protein), ELISA (enzyme-linked immunosorbent assay), IL-6 (interleukin-6), IL-8 (interleukin-8), PCT (procalcitonin), sFlt-1 (soluble fms-like tyrosine kinase-1), sTNFR1 (soluble tumor necrosis factor receptor), sTREM-1 (soluble triggering receptor expressed on myeloid cells 1).
