# Supplementary material for: Prognostic accuracy of biomarkers of immune and endothelial activation in Mozambican children hospitalized with pneumonia
Source: PLOS Glob Public Health. 2023 Feb 23;3(2):e0001553. doi: 10.1371/journal.pgph.0001553 (PMC10021812; doi:10.1371/journal.pgph.0001553)
Supplement: S2 Table — (DOCX) [file pgph.0001553.s002.docx]

**S2 Table. Biomarker concentrations in pneumonia cases by severity group**

| **Biomarker^a^** | **Pneumonia severe cases (n=220),**  **median (IQR)** | **Pneumonia non-severe cases (n=252),**  **median (IQR)** | **p-value^b^** |
| --- | --- | --- | --- |
| Angpt-2 | 4697.9 (2977.7, 7303.2) | 4045.3 (2718.6, 6652.3) | 0.101 |
| CRP | 64.2 (21.7, 149.1), n=216 | 84.2 (27.2, 167.7), n=245 | 0.076 |
| IL-6 | 30.8 (10.9, 145.8) | 35.2 (11.4, 83.0) | 0.529 |
| IL-8 | 18.5 (8.2, 49.2) | 13.8 (7.5, 35.1) | 0.046 |
| PCT | 1029.7 (325.2, 6457.8) | 1349.2 (286.8, 6781.2) | 0.973 |
| sFlt-1 | 220.1 (157.4, 334.9) | 202.2 (153.5, 287.4) | 0.079 |
| sTNFR1 | 2420.5 (1736.8, 3499.4) | 2559.0 (1783.8, 3944.1) | 0.402 |
| sTREM-1 | 200.9 (136.2, 308.7) | 196.7 (136.0, 279.2) | 0.565 |

**^a^** All in pg/mL, except CRP in µg/mL.

^b^ p-values were computed using the Mann-Whitney U test.

Abbreviations: Angpt-2 (angiopoietin-2), CRP (C-reactive protein), IL-6 (interleukin-6), IL-8 (interleukin-8), IQR (interquartile range), PCT (procalcitonin), sFlt-1 (soluble fms-like tyrosine kinase-1), sTNFR1 (soluble tumor necrosis factor receptor), sTREM-1 (soluble triggering receptor expressed on myeloid cells 1).
