# Supplementary material for: Prognostic accuracy of biomarkers of immune and endothelial activation in Mozambican children hospitalized with pneumonia
Source: PLOS Glob Public Health. 2023 Feb 23;3(2):e0001553. doi: 10.1371/journal.pgph.0001553 (PMC10021812; doi:10.1371/journal.pgph.0001553)
Supplement: S3 Table — (DOCX) [file pgph.0001553.s003.docx]

**S3 Table. Biomarker AUROCs for primary and secondary study outcomes**

| **In-hospital mortality** | | | | |
| --- | --- | --- | --- | --- |
| **Biomarker** | **n = 432^a^** | | **n = 421^b^** | |
|  | **AUROC (95% CI)** | **p-value** | **AUROC (95% CI)** | **p-value** |
| IL-8 | 0.877 (0.782, 0.972) | Reference^c^ | 0.868 (0.761, 0.975) | Reference^c^ |
| sFlt-1 | 0.832 (0.729, 0.935) | 0.473 | 0.855 (0.762, 0.948) | 0.835 |
| sTREM-1 | 0.822 (0.735, 0.908) | 0.177 | 0.794 (0.702, 0.886) | 0.106 |
| sTNFR1 | 0.736 (0.573, 0.898) | 0.004 | 0.703 (0.523, 0.883) | 0.002 |
| Angpt-2 | 0.598 (0.433, 0.762) | 0.001 | 0.545 (0.371, 0.719) | 0.001 |
| PCT | 0.590 (0.415, 0.764) | <0.001 | 0.555 (0.359, 0.751) | <0.001 |
| IL-6 | 0.587 (0.410, 0.764) | <0.001 | 0.587 (0.385, 0.788) | 0.001 |
| CRP | - | - | 0.402 (0.205, 0.600) | <0.001 |
| **28-day mortality** | | | | |
| **Biomarker** | **n = 309^a^** | | **n = 303^b^** | |
|  | **AUROC (95% CI)** | **p-value** | **AUROC (95% CI)** | **p-value** |
| IL-8 | 0.812 (0.716, 0.908) | Reference^c^ | 0.799 (0.696, 0.901) | Reference^c^ |
| sFlt-1 | 0.811 (0.712, 0.910) | 0.994 | 0.822 (0.723, 0.921) | 0.693 |
| sTREM-1 | 0.783 (0.684, 0.882) | 0.519 | 0.760 (0.655, 0.865) | 0.433 |
| sTNFR1 | 0.687 (0.546, 0.828) | 0.004 | 0.657 (0.507, 0.808) | 0.002 |
| Angpt-2 | 0.624 (0.490, 0.757) | 0.009 | 0.592 (0.453, 0.731) | 0.008 |
| PCT | 0.560 (0.425, 0.695) | <0.001 | 0.536 (0.391, 0.680) | <0.001 |
| IL-6 | 0.550 (0.397, 0.703) | <0.001 | 0.541 (0.374, 0.708) | <0.001 |
| CRP | - | - | 0.412 (0.257, 0.567) | <0.001 |
| **90-day mortality** | | | | |
| **Biomarker** | **n = 309^a^** | | **n = 303^b^** | |
|  | **AUROC (95% CI)** | **p-value** | **AUROC (95% CI)** | **p-value** |
| IL-8 | 0.799 (0.717, 0.880) | Reference^c^ | 0.788 (0.703, 0.873) | Reference^c^ |
| sTREM-1 | 0.757 (0.664, 0.850) | 0.389 | 0.737 (0.640, 0.835) | 0.329 |
| sFlt-1 | 0.742 (0.645, 0.839) | 0.293 | 0.744 (0.644, 0.844) | 0.429 |
| sTNFR1 | 0.645 (0.522, 0.768) | <0.001 | 0.619 (0.491, 0.747) | <0.001 |
| Angpt-2 | 0.601 (0.487, 0.715) | 0.003 | 0.575 (0.458, 0.691) | 0.002 |
| PCT | 0.519 (0.403, 0.634) | <0.001 | 0.496 (0.375, 0.617) | <0.001 |
| IL-6 | 0.514 (0.383, 0.645) | <0.001 | 0.503 (0.364, 0.643) | <0.001 |
| CRP | - | - | 0.380 (0.251, 0.509) | <0.001 |

^a^ First column AUROCs are derived excluding CRP from the analysis (11 individuals lacked CRP data, among whom 2 died during the hospital stay).

^b^ Second column AUROCs are derived using the subset of individuals with complete 8 biomarker data.

^c^ AUROCs were compared to IL-8 AUROC (reference), and p-values were computed using the algorithm suggested by DeLong *et al.*

Abbreviations: Angpt-2 (angiopoietin-2), AUROC (area under the receiver operating characteristic curve), CRP (C-reactive protein), IL-6 (interleukin-6), IL-8 (interleukin-8), PCT (procalcitonin), sFlt-1 (soluble fms-like tyrosine kinase-1), sTNFR1 (soluble tumor necrosis factor receptor), sTREM-1 (soluble triggering receptor expressed on myeloid cells 1).
