# Supplementary material for: Prognostic accuracy of biomarkers of immune and endothelial activation in Mozambican children hospitalized with pneumonia
Source: PLOS Glob Public Health. 2023 Feb 23;3(2):e0001553. doi: 10.1371/journal.pgph.0001553 (PMC10021812; doi:10.1371/journal.pgph.0001553)
Supplement: S4 Table — (DOCX) [file pgph.0001553.s004.docx]

**S4 Table. Biomarker concentrations in 28-day or 90-day deaths and survivors**

| **Biomarker^a^** | **28-day mortality** | | | **90-day mortality** | | |
| --- | --- | --- | --- | --- | --- | --- |
|  | **28-day deaths (n=22),**  **median (IQR)** | **28-day survivors (n=287), median (IQR)** | **p-value^b^** | **90-day deaths (n=28),**  **median (IQR)** | **90-day survivors (n=281), median (IQR)** | **p-value^b^** |
| Angpt-2 | 6191.0 (3361.5, 9231.3) | 4113.1 (2702.6, 6642.2) | 0.053 | 5534.1 (3235.5, 8980.4) | 4116.1 (2698.2, 6560.1) | 0.077 |
| CRP | 29.0 (6.4, 157.2), n=20 | 64.9 (22.9, 150.2), n=283 | 0.189 | 29.0 (5.7, 115.7), n=26 | 66.6 (23.3, 153.3), n=277 | 0.043 |
| IL-6 | 39.0 (6.3, 310.5) | 25.9 (9.7, 93.3) | 0.435 | 22.0 (7.1, 308.6) | 26.6 (9.7, 93.3) | 0.809 |
| IL-8 | 86.8 (24.4, 284.2) | 14.0 (6.8, 32.9) | <0.001 | 61.3 (23.4, 215.2) | 13.6 (6.8, 32.2) | <0.001 |
| PCT | 1660.7 (274.5, 22537.9) | 949.0 (266.2, 6495.0) | 0.347 | 846.4 (272.8, 10839.5) | 951.4 (267.3, 6570.6) | 0.747 |
| sFlt-1 | 452.7 (312.9, 848.3) | 201.7 (153.1, 287.0) | <0.001 | 360.5 (210.2, 603.4) | 202.2 (153.1, 291.7) | <0.001 |
| sTNFR1 | 4535.4 (2574.8, 7062.3) | 2316.4 (1657.0, 3480.9) | 0.004 | 3031.8 (2181.3, 6686.2) | 2314.7 (1657.0, 3494.0) | 0.011 |
| sTREM-1 | 344.8 (232.0, 512.5) | 188.3 (129.5, 265.1) | <0.001 | 304.2 (223.5, 493.0) | 187.5 (129.5, 261.4) | <0.001 |

**^a^** All in pg/mL, except CRP in µg/mL.

^b^ p-values were computed using the Mann-Whitney U test.

Abbreviations: Angpt-2 (angiopoietin-2), CRP (C-reactive protein), IL-6 (interleukin-6), IL-8 (interleukin-8), IQR (interquartile range), PCT (procalcitonin), sFlt-1 (soluble fms-like tyrosine kinase-1), sTNFR1 (soluble tumor necrosis factor receptor), sTREM-1 (soluble triggering receptor expressed on myeloid cells 1).
