# Supplementary material for: Prognostic accuracy of biomarkers of immune and endothelial activation in Mozambican children hospitalized with pneumonia
Source: PLOS Glob Public Health. 2023 Feb 23;3(2):e0001553. doi: 10.1371/journal.pgph.0001553 (PMC10021812; doi:10.1371/journal.pgph.0001553)
Supplement: S5 Table — (DOCX) [file pgph.0001553.s005.docx]

**S5 Table. Sensitivity analysis: Biomarker AUROCs for 28-day or 90-day deaths occurring only at post-discharge**

| **28-day mortality (post-discharge only)** | | | | |
| --- | --- | --- | --- | --- |
| **Biomarker** | **n = 294**^a^ | | **n = 290^b^** | |
|  | **AUROC (95% CI)** | **p-value** | **AUROC (95% CI)** | **p-value** |
| sFlt-1 | 0.744 (0.523, 0.965) | Reference^c^ | 0.744 (0.523, 0.964) | Reference^c^ |
| sTREM-1 | 0.686 (0.447, 0.925) | 0.742 | 0.684 (0.445, 0.923) | 0.738 |
| IL-8 | 0.659 (0.473, 0.845) | 0.506 | 0.660 (0.475, 0.844) | 0.510 |
| Angpt-2 | 0.656 (0.417, 0.894) | 0.320 | 0.655 (0.417, 0.892) | 0.317 |
| sTNFR1 | 0.559 (0.290, 0.828) | 0.269 | 0.557 (0.287, 0.827) | 0.265 |
| PCT | 0.485 (0.228, 0.712) | 0.064 | 0.482 (0.255, 0.709) | 0.061 |
| IL-6 | 0.423 (0.128, 0.718) | 0.013 | 0.421 (0.125, 0.716) | 0.013 |
| CRP | - | - | 0.409 (0.149, 0.669) | 0.001 |
| **90-day mortality (post-discharge only)** | | | | |
| **Biomarker** | **n = 294**^a^ | | **n = 290^b^** | |
|  | **AUROC (95% CI)** | **p-value** | **AUROC (95% CI)** | **p-value** |
| IL-8 | 0.700 (0.584, 0.816) | Reference^c^ | 0.701 (0.586, 0.816) | Reference^c^ |
| sTREM-1 | 0.672 (0.512, 0.833) | 0.759 | 0.671 (0.510, 0.832) | 0.739 |
| sFlt-1 | 0.628 (0.478, 0.777) | 0.430 | 0.626 (0.476, 0.776) | 0.415 |
| Angpt-2 | 0.593 (0.437, 0.749) | 0.259 | 0.592 (0.436, 0.748) | 0.250 |
| sTNFR1 | 0.529 (0.359, 0.698) | 0.003 | 0.527 (0.356, 0.697) | 0.003 |
| PCT | 0.433 (0.289, 0.577) | <0.001 | 0.429 (0.285, 0.573) | <0.001 |
| IL-6 | 0.406 (0.224, 0.588) | <0.001 | 0.402 (0.220, 0.585) | <0.001 |
| CRP | - | - | 0.350 (0.183, 0.516) | <0.001 |

^a^ First column AUROCs are derived excluding CRP from the analysis (11 individuals lacked CRP data, among whom 2 died during the hospital stay).

^b^ Second column AUROCs are derived using the subset of individuals with complete 8 biomarker data.

^c^ AUROCs were compared to IL-8 AUROC (reference), and p-values were computed using the algorithm suggested by DeLong *et al.*

Abbreviations: Angpt-2 (angiopoietin-2), AUROC (area under the receiver operating characteristic curve), CRP (C-reactive protein), IL-6 (interleukin-6), IL-8 (interleukin-8), PCT (procalcitonin), sFlt-1 (soluble fms-like tyrosine kinase-1), sTNFR1 (soluble tumor necrosis factor receptor), sTREM-1 (soluble triggering receptor expressed on myeloid cells 1).
