# Supplementary material for: Prognostic accuracy of biomarkers of immune and endothelial activation in Mozambican children hospitalized with pneumonia
Source: PLOS Glob Public Health. 2023 Feb 23;3(2):e0001553. doi: 10.1371/journal.pgph.0001553 (PMC10021812; doi:10.1371/journal.pgph.0001553)
Supplement: S6 Table — (DOCX) [file pgph.0001553.s006.docx]

**S6 Table. Demographic and clinical characteristics associated with in-hospital mortality in pneumonia cases**

| **Variable^a^** | **Total pneumonia cases with a valid primary outcome (n=432)** | **In-hospital deaths (n=15)** | **OR (95% CI)**^b^ | **p-value** |
| --- | --- | --- | --- | --- |
| **Sex** |  |  |  |  |
| Male | 221 | 9 (4.1) | 1 | 0.484 |
| Female | 211 | 6 (2.8) | 0.69 (0.24, 1.97) |  |
| **Age** |  |  |  |  |
| <2 months | 23 | 1 (4.4) | 1 | 0.606 |
| 2 to 11 months | 162 | 7 (4.4) | 0.99 (0.12, 8.46) |  |
| 12 to 59 months | 214 | 5 (2.4) | 0.53 (0.06, 4.71) |  |
| 5 to <10 years | 33 | 2 (6.1) | 1.42 (0.12, 16.64) |  |
| Median (months) | 14.8 [6.3, 27.5] | 10.5 [3.0, 24.1] | 1.00 (0.97, 1.02) | 0.710 |
| **WAZ^c^** |  |  |  |  |
| >-1 | 160 | 1 (0.6) | 1 | 0.001 |
| -1 to -3 | 198 | 6 (3.0) | 4.97 (0.59, 41.70) |  |
| <-3 | 74 | 8 (10.8) | 19.27 (2.36, 157.16) |  |
| Median | -1.5 [-2.6, -0.5] | -3.4 [-4.2, -1.6] | 0.67 (0.53, 0.85) | 0.002 |
| **MUAC (cm)** | 14.0 [13.0, 15.0], n=424 | 12.0 [10.0, 13.0] | 0.53 (0.38, 0.73) | <0.001 |
| **HIV status** |  |  |  |  |
| Negative | 353 | 8 (2.3) | 1 | 0.010 |
| Positive | 79 | 7 (8.9) | 4.19 (1.47, 11.93) |  |
| **Malaria parasitemia** |  |  |  |  |
| Negative | 350 | 14 (4.0) | 1 | 0.166 |
| Positive | 82 | 1 (1.2) | 0.30 (0.04, 2.29) |  |
| **Invasive bacterial disease^d^** |  |  |  |  |
| Negative | 380 | 8 (2.1) | 1 | 0.001 |
| Positive | 52 | 7 (13.5) | 7.23 (2.50, 20.89) |  |
| **Viral respiratory infection^e^**, n=419 |  |  |  |  |
| Negative | 143 | 7 (4.9) | 1 | 0.214 |
| Positive | 276 | 7 (2.5) | 0.51 (0.17, 1.47) |  |
| **Axillary temperature** | 38.1 [37.1, 39.1] | 37.6 [36.5, 38.7] | 0.69 (0.44, 1.07) | 0.092 |
| **Fever (≥37.5ºC, axillary temperature)** |  |  |  |  |
| No | 134 | 7 (5.2) | 1 | 0.197 |
| Yes | 298 | 8 (2.7) | 0.50 (0.18, 1.41) |  |
| **SpO_2_ (units decreased from 100%)** | 3 [2, 4], n=412 | 3 [3, 9] | 1.19 (1.02, 1.41) | 0.049 |
| **Hypoxemia (SpO_2_<90%)**, n=412 |  |  |  |  |
| No | 403 | 14 (3.5) | 1 | 0.324 |
| Yes | 9 | 1 (11.1) | 3.47 (0.41, 29.70) |  |
| **Cyanosis** |  |  |  |  |
| No | 417 | 14 (3.4) | 1 | 0.538 |
| Yes | 15 | 1 (6.7) | 2.06 (0.25, 16.75) |  |
| **Respiratory rate** | 56 [49, 62] | 54 [44, 60] | 0.98 (0.93, 1.03) | 0.320 |
| **Low chest wall indrawing** |  |  |  |  |
| No | 143 | 4 (2.8) | 1 | 0.583 |
| Yes | 289 | 11 (3.8) | 1.38 (0.43, 4.40) |  |
| **Nasal flaring** |  |  |  |  |
| No | 227 | 8 (3.5) | 1 | 0.950 |
| Yes | 205 | 7 (3.4) | 0.97 (0.34, 2.72) |  |
| **Deep breathing** |  |  |  |  |
| No | 385 | 12 (3.1) | 1 | 0.292 |
| Yes | 47 | 3 (6.4) | 2.12 (0.58, 7.80) |  |
| **Grunting** |  |  |  |  |
| No | 384 | 12 (3.1) | 1 | 0.307 |
| Yes | 48 | 3 (6.3) | 2.07 (0.56, 7.60) |  |
| **Wheezing** |  |  |  |  |
| No | 331 | 13 (3.9) | 1 | 0.320 |
| Yes | 101 | 2 (2.0) | 0.49 (0.11, 2.23) |  |
| **Crackles** |  |  |  |  |
| No | 190 | 5 (2.6) | 1 | 0.392 |
| Yes | 242 | 10 (4.1) | 1.59 (0.54, 4.75) |  |
| **Ronchi** |  |  |  |  |
| No | 281 | 11 (3.9) | 1 | 0.484 |
| Yes | 151 | 4 (2.7) | 0.67 (0.21, 2.13) |  |
| **Inspiratory stridor** |  |  |  |  |
| No | 414 | 15 (3.6) | - | - |
| Yes | 18 | 0 (0) | - |  |
| **Altered consciousness (BCS<5)** |  |  |  |  |
| No | 416 | 14 (3.4) | 1 | 0.575 |
| Yes | 16 | 1 (6.3) | 1.91 (0.24, 15.53) |  |
| **Coma (BCS<3)** |  |  |  |  |
| No | 431 | 15 (3.5) | - | - |
| Yes | 1 | 0 (0) | - |  |
| **Prostration** |  |  |  |  |
| No | 395 | 13 (3.3) | 1 | 0.530 |
| Yes | 37 | 2 (5.4) | 1.68 (0.36, 7.74) |  |
| **Convulsions**, n=423 |  |  |  |  |
| No | 398 | 14 (3.5) | 1 | 0.901 |
| Yes | 25 | 1 (4.0) | 1.14 (0.14, 9.06) |  |
| **Unable to drink/breastfeed**, n=422 |  |  |  |  |
| No | 402 | 13 (3.2) | 1 | 0.183 |
| Yes | 20 | 2 (10.0) | 3.32 (0.70, 15.85) |  |
| **Dehydration**, n=423 |  |  |  |  |
| No | 396 | 10 (2.5) | 1 | 0.001 |
| Yes | 27 | 5 (18.5) | 8.77 (2.76, 27.88) |  |
| **Radiological findings**, n=375 |  |  |  |  |
| Normal | 138 | 2 (1.5) | 1 | 0.765 |
| Other infiltrates | 85 | 2 (2.4) | 1.64 (0.23, 11.85) |  |
| Endpoint pneumonia | 152 | 4 (2.6) | 1.84 (0.33, 10.19) |  |
| **Glucose (mmol/L)** | 6.1 [5.2, 7.0], n=390 | 5.9 [4.3, 7.7], n=13 | 1.12 (0.84, 1.50) | 0.454 |
| **Hematocrit (%)** | 27.4 [23.1, 31.1], n=430 | 24.3 [22.9, 26.8] | 0.96 (0.88, 1.04) | 0.300 |
| **WBC count** | 14.2 [10.1, 20.4], n=409 | 14.6 [12.2, 21.1] | 1.01 (0.95, 1.06) | 0.814 |
| **WHO severity definition** |  |  |  |  |
| No | 229 | 5 (2.2) | 1 | 0.118 |
| Yes | 203 | 10 (4.9) | 2.32 (0.78, 6.91) |  |
| **LODS** | 0 [0, 0] | 0 [0, 1] | 1.64 (0.70, 3.85) | 0.295 |
| **RISC-Malawi score** | 1 [0, 4], n=405 | 5 [2, 8] | 1.38 (1.18, 1.61) | <0.001 |
| **Previous admissions for pneumonia** |  |  |  |  |
| No | 417 | 12 (2.9) | 1 | 0.011 |
| Yes | 15 | 3 (20.0) | 8.44 (2.10, 33.85) |  |

^a^ Data presented as median [interquartile range] or frequency (percent) as appropriate. Number of subjects indicated if presence of missing values.

^b^ ORs, 95% CIs, and p-values are from univariable logistic regression models.

^c^ Calculated using the LMS method and the 2000 US CDC Growth Reference data.

^d^ Defined as the detection of a non-contaminating bacterial organism in blood, cerebrospinal fluid, and/or pleural fluid through culture or PCR analysis of dried blood spots for *Streptococcus pneumoniae* and *Haemophilus influenzae* type B.

^e^ At least one virus detected in nasopharyngeal aspirate. PCR primers targeted adenovirus, bocavirus, coronavirus (229E and OC43), enterovirus, influenza virus (A, B, and C), metapneumovirus, parainfluenza virus (1, 2, 3, and 4), respiratory syncytial virus (A and B), and rhinovirus.

Abbreviations: BCS (Blantyre Coma Scale), CI (confidence interval), HIV (human immunodeficiency virus), LODS (Lambaréné Organ Dysfunction Score), MUAC (mid-upper arm circumference), OR (odds ratio), RISC-Malawi (Respiratory Index of Severity in Children-Malawi), WAZ (weight-for-age z-score), WBC (white blood cell), WHO (World Health Organization).
