# Supplementary material for: Prognostic accuracy of biomarkers of immune and endothelial activation in Mozambican children hospitalized with pneumonia
Source: PLOS Glob Public Health. 2023 Feb 23;3(2):e0001553. doi: 10.1371/journal.pgph.0001553 (PMC10021812; doi:10.1371/journal.pgph.0001553)
Supplement: S7 Table — (DOCX) [file pgph.0001553.s007.docx]

**S7 Table. Biomarker performance estimators to identify in-hospital mortality using cut-offs based on the Younden Index**

| **Biomarker** | **Cut-off** | **Sensitivity (95% CI)** | **Specificity**  **(95% CI)** | **LR+**  **(95% CI)** | **LR-**  **(95% CI)** | **PPV**  **(95% CI)** | **NPV**  **(95% CI)** |
| --- | --- | --- | --- | --- | --- | --- | --- |
| IL-8 | ≥59.6 pg/mL | 80.0 (51.9, 95.7) | 86.8 (83.2, 89.9) | 6.07 (4.26, 8.63) | 0.23 (0.08, 0.63) | 17.9 (9.6, 29.2) | 99.2 (97.6, 99.8) |
| sFlt-1 | ≥312.9 pg/mL | 80.0 (51.9, 95.7) | 78.2 (73.9, 82.0) | 3.67 (2.68, 5.01) | 0.26 (0.09, 0.70) | 11.7 (6.2, 19.5) | 99.1 (97.4, 99.8) |
| sTREM-1 | ≥232.0 pg/mL | 86.7 (59.5, 98.3) | 66.9 (62.2, 71.4) | 2.62 (2.06, 3.33) | 0.20 (0.05, 0.73) | 8.6 (4.7, 14.3) | 99.3 (97.5, 99.9) |
| sTNFR1 | ≥4613.2 pg/mL | 60.0 (32.3, 83.7) | 86.1 (82.4, 89.3) | 4.31 (2.68, 6.95) | 0.46 (0.25, 0.86) | 13.4 (6.3, 24.0) | 98.4 (96.5, 99.4) |
| Angpt-2 | ≥4782.3 pg/mL | 66.7 (38.4, 88.2) | 57.1 (52.2, 61.9) | 1.55 (1.07, 2.26) | 0.58 (0.28, 1.20) | 5.3 (2.6, 9.5) | 97.9 (95.3, 99.3) |
| PCT | ≥6420.6 pg/mL | 53.3 (26.6, 78.7) | 77.0 (72.6, 80.9) | 2.32 (1.40, 3.84) | 0.61 (0.35, 1.04) | 7.7 (3.4, 14.6) | 97.9 (95.7, 99.1) |
| IL-6 | ≥272.7 pg/mL | 40.0 (16.3, 67.7) | 87.8 (84.2, 90.8) | 3.27 (1.67, 6.40) | 0.68 (0.45, 1.03) | 10.5 (4.0, 21.5) | 97.6 (95.5, 98.9) |
| CRP | ≥195.7 µg/mL | 30.8 (9.1, 61.4) | 82.8 (78.8, 86.4) | 1.79 (0.77, 4.17) | 0.84 (0.58, 1.20) | 5.4 (1.5, 13.3) | 97.4 (95.1, 98.8) |

Abbreviations: Angpt-2 (angiopoietin-2), CRP (C-reactive protein), IL-6 (interleukin-6), IL-8 (interleukin-8), LR+ (positive likelihood ratio), LR- (negative likelihood ratio), NPV (negative predictive value), PCT (procalcitonin), PPV (positive predictive value), sFlt-1 (soluble fms-like tyrosine kinase-1), sTNFR1 (soluble tumor necrosis factor receptor), sTREM-1 (soluble triggering receptor expressed on myeloid cells 1).
