# Supplementary material for: Prognostic accuracy of biomarkers of immune and endothelial activation in Mozambican children hospitalized with pneumonia
Source: PLOS Glob Public Health. 2023 Feb 23;3(2):e0001553. doi: 10.1371/journal.pgph.0001553 (PMC10021812; doi:10.1371/journal.pgph.0001553)
Supplement: S1 Fig — (DOCX) [file pgph.0001553.s008.docx]

**S1 Fig. Biomarker Spearman’s correlation coefficients in pneumonia cases**

**
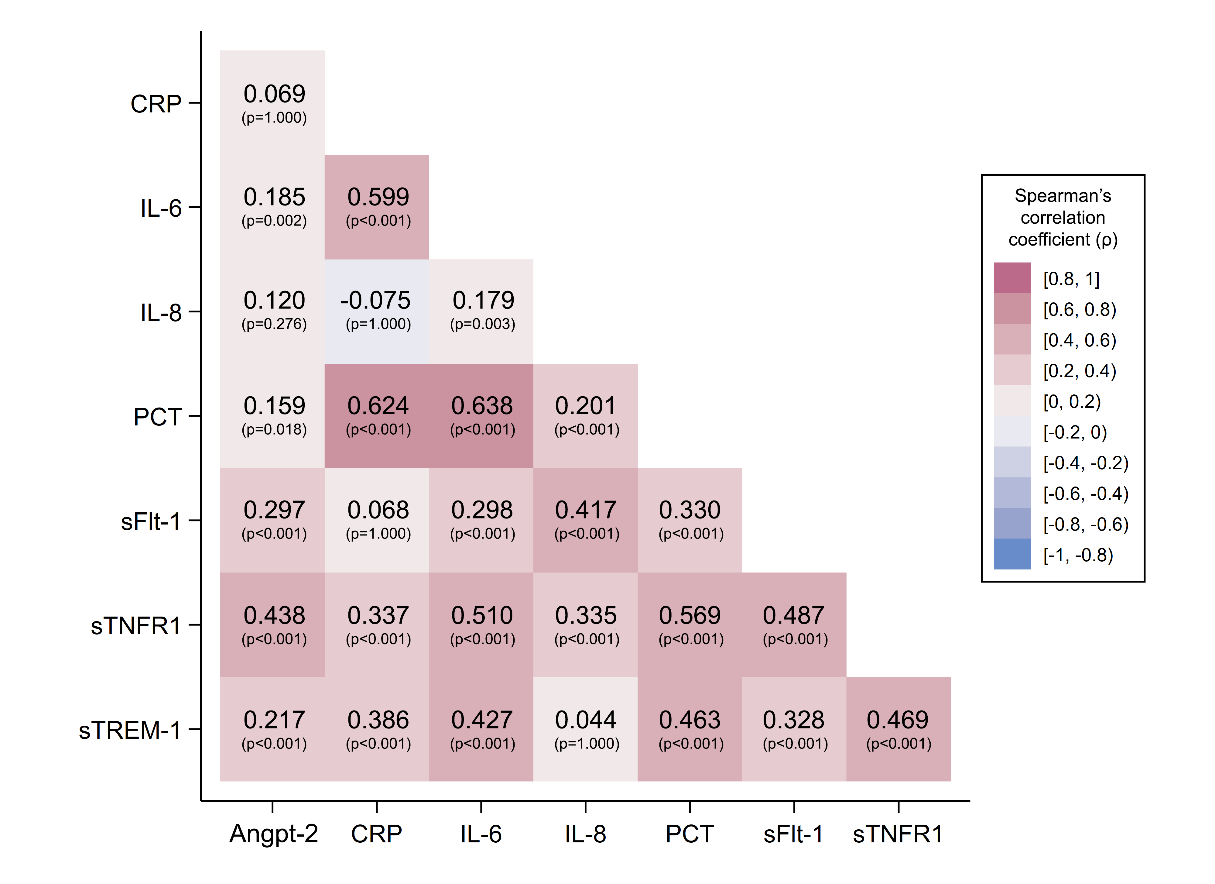
**

* p-values were adjusted using the Bonferroni correction.

Abbreviations: Angpt-2 (angiopoietin-2), CRP (C-reactive protein), IL-6 (interleukin-6), IL-8 (interleukin-8), PCT (procalcitonin), sFlt-1 (soluble fms-like tyrosine kinase-1), sTNFR1 (soluble tumor necrosis factor receptor), sTREM-1 (soluble triggering receptor expressed on myeloid cells 1).
